# Effect of lymph node dissection numbers after conversion immunochemotherapy on the survival of gastric cancer patients: a multi-center retrospective study

**DOI:** 10.3389/fimmu.2026.1862248

**Published:** 2026-07-15

**Authors:** Mengjie Liang, Long Qian, Zhengyang Yang, Yiwen Sun, Shichao Ai, Peng Song, Qianyu Wang, Feng Sun, Xingzhou Wang, Xiaofeng Lu, Meng Wang, Ji Miao, Jie Ding, Junfeng Du, Wenxian Guan, Xiaofei Shen

**Affiliations:** 1 Division of Gastric Surgery, Department of General Surgery, Nanjing Drum Tower Hospital, the Affiliated Hospital of Nanjing University Medical School, Nanjing, China; 2 MOE Key Laboratory of Model Animal for Disease Study, Nanjing Drum Tower Hospital, Affiliated Hospital of Nanjing University Medical School, Nanjing, China; 3 Department of General Surgery, Beijing Friendship Hospital, Capital Medical University, State Key Lab of Digestive Health, National Clinical Research Center for Digestive Diseases, Beijing, China; 4 Department of General Surgery, Peking Union Medical College Hospital, Chinese Academy of Medical Sciences, Beijing, China; 5 Department of General Surgery, The 7th Medical Center, Chinese PLA General Hospital, Beijing, China

**Keywords:** immunochemotherapy, locally advanced gastric cancer, lymph node dissection, lymph node metastasis, prognosis

## Abstract

**Objective:**

To investigate the influence of lymph node dissection on the prognosis of unresectable locally advanced gastric cancer (LAGC) after conversion immunochemotherapy (CICT).

**Methods:**

A total of 287 patients, including 227 from Nanjing Drum Tower Hospital and an external validation cohort of 60 patients (15 from Peking Union Medical College Hospital, 20 from Beijing Friendship Hospital, and 25 from PLA General Hospital) with pathologically diagnosed unresectable primary LAGC (cT3~4N+) underwent radical gastrectomy after multidisciplinary evaluation conversion immunochemotherapy (CICT) were enrolled, and their clinical data were collected. According to the pathological data of patients’ baseline data set, a clinical prediction model of patients’ overall survival (OS) and progression-free survival (PFS) was established, and based on COX regression model, the critical value of the number of lymph node dissection was determined by restricted cubic spline (RCS) analysis.

**Results:**

Univariate and multivariate COX regression analysis showed that lymph node metastasis (ypN+) was an independent risk factor for OS (P = 0.030) and PFS (P = 0.002). According to the results of COX regression analysis, the prediction model was established. RCS curve showed that the critical number of lymph node dissection was 21 and 28. Patients with 21–28 lymph node dissection had the best OS and PFS. Compared with the patients with 21–28 lymph nodes dissection, the patients with less than 21 lymph nodes dissection had worse OS (P<0.001) and PFS (P = 0.002). Those with more than 28 lymph nodes isolated had worse OS(P = 0.043) and PFS (P = 0.027). The PFS of patients with more than 28 lymph nodes dissection was better than the patients with less than 21lymph nodes dissection (P = 0.031). Although the difference was not statistically significant, the total OS of patients with more than 28 lymph nodes dissection was still higher than that of patients with less than 21 lymph nodes dissection (P = 0.077).

**Conclusion:**

Among patients after CICT, lymph node metastasis (ypN+) is an independent risk factor for the prognosis of patients. The survival benefit of patients with less than 21 lymph node dissection is significantly reduced, while those with more than 28 lymph node dissection are less than 21–28 lymph node dissection.

## Highlights

- This study focused on unmet clinical needs: Targets two core unresolved issues in post-conversion immunochemotherapy (CICT) lymph node dissection strategies for unresectable local advanced gastric cancer (LAGC) patients namely the prognostic value of pathological nodal (ypN) stage post-immunotherapy and the impact of harvested lymph node (LN) count on survival.
- First multicenter retrospective study confirmed that ypN+ (post-CICT lymph node metastasis) is an independent risk factor for overall survival (OS) and progression-free survival (PFS) in this specific patient population.
- Defined optimal LN dissection range: Identified 21–28 as the optimal number of dissected lymph nodes for unresectable LAGC patients after preoperative CICT, with both insufficient and excessive dissection compromising surgical efficacy.
- Clinical guideline implication: Provided high-value real-world evidence to support the future development of clinical guidelines for lymph node dissection in the era of gastric cancer immunotherapy.

## Introduction

Gastric cancer (GC) is a globally prevalent malignancy and a leading cause of cancer-related deaths (1), imposing a substantial cancer burden in China (2). Most patients are diagnosed with locally advanced gastric cancer (LAGC) initially. Current clinical studies on immune checkpoint inhibitors (ICIs), represented by programmed death-1 (PD-1) inhibitors, have demonstrated that preoperative conversion immunochemotherapy (CICT) yields more significant survival benefits compared to preoperative chemotherapy alone, gradually becoming the first-line option for preoperative conversion therapy in LAGC.

The stomach is surrounded by a rich lymphatic system, and lymph node (LN) status is a crucial prognostic factor for GC patients. Antigen presentation to tumor-draining lymph nodes is a vital step in establishing the body’s immune surveillance against tumors (3), and the metastatic status of lymph nodes remains a significant factor influencing patients’ survival (4). Non-metastatic lymph nodes are rich in progenitor exhausted CD8+ T cells (CD8+Tpex) that are clonally related to terminally exhausted cells in the tumor, suggesting a decrease in CD8+Tpex in metastatic lymph nodes. This indicates that LNs, especially non-metastatic LNs, function in anti-tumor immunotherapy by continuously supplying anti-tumor-active CD8+ T cells to the tumor area (5). Lymph node dissection is a critical step in radical surgery for gastric cancer and a focal point of clinical controversy (6). On one hand, lymph node dissection can provide a complete basis for postoperative N staging and may reduce the risk of postoperative recurrence and metastasis caused by lymph node micro-metastasis. Current guidelines recommend the number of dissected nodes should be ≥16, and preferably >30 (4). However, excessive lymph node dissection may damage the reservoir of anti-tumor immune cells in the LNs, reducing survival benefits (7). With the deepening understanding and application of ICIs, new perspectives on lymph nodes have emerged, and extensive lymph node dissection may reduce the efficacy brought by ICIs. Studies have already shown that excessive lymph node dissection reduces the therapeutic benefits of ICIs in non-small cell lung cancer (8), biliary tract cancer (9) and colorectal cancer (10, 11). Consequently, some scholars have suggested that tumor-draining lymph nodes should be appropriately preserved (12). However, there is still a need to coordinate immunochemotherapy with standard therapy for rational design.

Therefore, this study aimed to conduct a retrospective analysis of LAGC patients who received CICT combined with surgical treatment, exploring independent prognostic risk factors and the impact of lymph node dissection number on prognosis, so as to provide evidence-based support for individualized lymph node dissection.

## 1 Materials andmethods

### 1.1 Study subjects

This retrospective observational study included patients meeting the following inclusion criteria: (1) 18–80 years old; (2) Pathologically confirmed gastric cancer; (3) Assessed as unresectable locally advanced gastric cancer (cT3-4N+) by the gastric surgery, oncology, and radiology departments of participating institutions (Nanjing Drum Tower Hospital, Peking Union Medical College Hospital, Beijing Friendship Hospital affiliated with Capital Medical University, and the Seventh Medical Center of the Chinese PLA General Hospital); (4) Completed a certain cycle of CICT (conversion immunochemotherapy) followed by radical total gastrectomy (to control for the impact of dissection scope) after multidisciplinary evaluation; (5) Complete postoperative pathological data; (6) D2 lymph node dissection with ≥16 lymph nodes dissected; (7) Complete clinical and follow-up data; (8) No autoimmune diseases. Exclusion criteria were: (1) M1 status before conversion therapy; (2) Remnant gastric cancer; (3) Concomitant other tumors; (4) Unplanned surgery during conversion therapy; (5) Non-R0 resection. This study was approved by the Ethics Committee of Nanjing Drum Tower Hospital, the Affiliated Hospital of Nanjing University Medical School (Ethics approval number: 2025-0099-01), Beijing Friendship Hospital (2021-P2-318-01), the PLAGH(S2025-047-02), and PUMCH (I-25PJ0636).

### 1.2 Treatment of CICT

The primary CICT regimen was a PD-1 inhibitor combined with SOX (Tegafur gimeracil oteracil potassium + Oxaliplatin). All patients underwent radical total gastrectomy with D2 lymph node dissection.

### 1.3 Data collection and follow-up

Patients’ clinical data were obtained from the electronic medical record system, with specific details shown in **Table 1**, including: (1) Basic patients’ information (sex, age, body mass index (BMI), etc.); (2) Surgical information (operation time, surgical method, etc.); (3) Pathological data, postoperative pathology strictly followed the AJCC 8th edition guidelines (13)(Both preoperative and postoperative pathological evaluations were performed by the pathology departments of the respective centers, and the results were interpreted and reviewed by two or more senior pathologists. All dissected lymph nodes were assessed to obtain accurate lymph node information and ypN staging), collecting postoperative staging, number of dissected lymph nodes, etc. TRG grading was defined as follows: TRG0, no residual tumor cells; TRG1, <10% residual tumor cells; TRG2, 10%~50% residual tumor cells; TRG3, >50% residual tumor cells. Because some patients had complete tumor regression (TRG 0), tumor/tumor bed size was used for description. Based on the anatomical location of the tumor bed/tumor, it was divided into upper stomach (cardia, fundus), middle stomach (body), lower stomach (angle to pylorus[Prior to undergoing CICT, these patients had cross-segment gastric cancer involving the body and lower part of the stomach; they therefore underwent total gastrectomy as a curative treatment for gastric cancer.]), and cross-regional (upper-middle, middle-lower, total stomach); (4) and follow-up information, including overall survival (OS): time from radical gastrectomy to death from any cause or the follow-up deadline; progression-free survival (PFS): interval from radical gastrectomy to the date of tumor recurrence/metastasis. If no recurrence/metastasis occurred by the last follow-up, the time of the last follow-up or death was used as the endpoint. The follow-up deadline was September 30, 2025.

**Table 1:** Baseline data of 287 patients and baseline data of the training cohort (n=158), internal validation cohort (n=69), and external validation cohort (n=60).

Variables	Total (n = 287)	Internal validation cohort (n = 69)	External validation cohort (n=60)	Training cohort (n=158)	Statistic	*P*
Gender, n (%)					χ²=0.78	0.676
Female	60 (20.91)	16 (23.19)	14 (23.33)	30 (18.99)		
Male	227 (79.09)	53 (76.81)	46 (76.67)	128 (81.01)		
Age/years (x ± s)	64.66 ± 9.32	63.78 ± 9.78	64.02 ± 9.01	65.29 ± 9.23	F=0.81	0.446
BMI/Kg·m-2, (x ± s)	22.48 ± 3.05	22.00 ± 2.91	22.17 ± 3.02	22.81 ± 3.09	F=2.12	0.122
Diabetes, n (%)					χ²=0.13	0.936
No	237 (82.58)	56 (81.16)	50 (83.33)	131 (82.91)		
Yes	50 (17.42)	13 (18.84)	10 (16.67)	27 (17.09)		
Hypertension, n (%)					χ²=0.55	0.758
No	215 (74.91)	54 (78.26)	44 (73.33)	117 (74.05)		
Yes	72 (25.09)	15 (21.74)	16 (26.67)	41 (25.95)		
Surgical method, n (%)					χ²=0.04	0.982
Open surgery	257 (89.55)	62 (89.86)	54 (90.00)	141 (89.24)		
Laparoscopic surgery	30 (10.45)	7 (10.14)	6 (10.00)	17 (10.76)		
Intraoperative blood loss/ml (x ± s)	144.81 ± 125.02	132.03 ± 113.65	130.17 ± 113.08	155.95 ± 133.39	F=1.40	0.247
Operation time/min, (x ± s)	198.17 ± 47.86	192.93 ± 46.04	193.65 ± 41.80	202.15 ± 50.60	F=1.22	0.295
Tumor location, n (%)					-	0.914
Middle	70 (24.39)	15 (21.74)	13 (21.67)	42 (26.58)		
Lower	32 (11.15)	7 (10.14)	6 (10.00)	19 (12.03)		
Upper	150 (52.26)	39 (56.52)	33 (55.00)	78 (49.37)		
Upper-Middle	14 (4.88)	3 (4.35)	3 (5.00)	8 (5.06)		
Middle-Lower	16 (5.57)	3 (4.35)	3 (5.00)	10 (6.33)		
Total	5 (1.74)	2 (2.90)	2 (3.33)	1 (0.63)		
Histological type, n(%)					χ²=8.09	0.424
Not applicable*	79 (27.53)	19 (27.54)	18 (30.00)	42 (26.58)		
Tubular adenocarcinoma	97 (33.80)	20 (28.99)	16 (26.67)	61 (38.61)		
PCC (incl. SRCC*)	47 (16.38)	13 (18.84)	13 (21.67)	21 (13.29)		
Mixed adenocarcinoma	38 (13.24)	11 (15.94)	10 (16.67)	17 (10.76)		
Other rare tumors*	26 (9.06)	6 (8.70)	3 (5.00)	17 (10.76)		
Mucinous/colloid adenocarcinoma	9 (3.14)	2 (2.90)	1 (1.67)	6 (3.80)		
Hepatoid adenocarcinoma	4 (1.39)	1 (1.45)	0 (0)	3 (1.90)		
Micropapillary adenocarcinoma	1 (0.35)	0 (0)	0 (0)	1 (0.63)		
MiNEN*	3 (1.05)	0 (0)	1 (1.67)	2 (1.27)		
Neuroendocrine carcinoma	3 (1.05)	1 (1.45)	1 (1.67)	1 (0.63)		
EBV-associated gastric cancer*	3 (1.05)	1 (1.45)	0 (0)	2 (1.27)		
Medullary carcinoma with lymphoid stroma	2 (0.70)	1 (1.45)	0 (0)	1 (0.63)		
TRG, n (%)*					χ²=9.32	0.156
0	64 (22.30)	18 (26.09)	16 (26.67)	30 (18.99)		
1	73 (25.44)	17 (24.64)	14 (23.33)	42 (26.58)		
2	92 (32.06)	24 (34.78)	23 (38.33)	45 (28.48)		
3	58 (20.21)	10 (14.49)	7 (11.67)	41 (25.95)		
AJCC tumor stage, n(%)					χ²=5.64	0.688
pCR*	60 (20.91)	17 (24.64)	15 (25.00)	28 (17.72)		
Stage I	53 (18.47)	14 (20.29)	14 (23.33)	25 (15.82)		
Stage II	77 (26.83)	18 (26.09)	13 (21.67)	46 (29.11)		
Stage III	93 (32.40)	19 (27.54)	17 (28.33)	57 (36.08)		
ypT0N+	4 (1.39)	1 (1.45)	1 (1.67)	2 (1.27)		
Tumor/tumor bed max. diameter, n (%)					χ²=0.29	0.863
<5cm	198 (68.99)	47 (68.12)	40 (66.67)	111 (70.25)		
≥5cm	89 (31.01)	22 (31.88)	20 (33.33)	47 (29.75)		
T stage, n (%)					χ²=3.91	0.865
ypT0	64 (22.30)	18 (26.09)	16 (26.67)	30 (18.99)		
ypT1	39 (13.59)	10 (14.49)	10 (16.67)	19 (12.03)		
ypT2	33 (11.50)	7 (10.14)	6 (10.00)	20 (12.66)		
ypT3	118 (41.11)	27 (39.13)	22 (36.67)	69 (43.67)		
ypT4	33 (11.50)	7 (10.14)	6 (10.00)	20 (12.66)		
Lymph node metastasis (ypN+), n (%)					χ²=2.20	0.333
No	162 (56.45)	42 (60.87)	37 (61.67)	83 (52.53)		
Yes	125 (43.55)	27 (39.13)	23 (38.33)	75 (47.47)		
Lauren classification, n (%)					χ²=1.72	0.944
Not applicable*	99 (34.49)	24 (34.78)	22 (36.67)	53 (33.54)		
Intestinal	104 (36.24)	23 (33.33)	19 (31.67)	62 (39.24)		
Diffuse	47 (16.38)	12 (17.39)	10 (16.67)	25 (15.82)		
Mixed	37 (12.89)	10 (14.49)	9 (15.00)	18 (11.39)		
Differentiation grade, n (%)					χ²=1.39	0.967
Not applicable*	77 (26.83)	20 (28.99)	18 (30.00)	39 (24.68)		
Well differentiated (G1)	13 (4.53)	3 (4.35)	2 (3.33)	8 (5.06)		
Moderately differentiated (G2)	94 (32.75)	21 (30.43)	18 (30.00)	55 (34.81)		
Poorly differentiated (G3)	103 (35.89)	25 (36.23)	22 (36.67)	56 (35.44)		
Vascular invasion, n (%)					χ²=3.90	0.142
No	211 (73.52)	57 (82.61)	43 (71.67)	111 (70.25)		
Yes	76 (26.48)	12 (17.39)	17 (28.33)	47 (29.75)		
Neural invasion, n (%)					χ²=0.16	0.922
No	173 (60.28)	43 (62.32)	36 (60.00)	94 (59.49)		
Yes	114 (39.72)	26 (37.68)	24 (40.00)	64 (40.51)		
Median (month, 95% CI)						
follow-up time	38.20(30.97-40.30)	31.83 (29.83 - 38.93)	41.60 (38.80 - 48.00)	38.90(28.97 - 46.80)	-	0.372
OS	49.40(46.43-NA)	NA (38.63 - NA)	48.90 (37.10 - NA)	49.33 (38.33 - NA)	-	0.890
PFS	37.60(34.03-NA)	NA (23.63 - NA)	36.70 (33.07 - 40.23)	NA (26.43 - NA)	-	0.573

F, ANOVA; χ², Chi-square test; -: Fisher exact; SD, standard deviation.

*Not applicable: Included patients received preoperative CICT, and those with significant tumor regression reached pCR or had minimal residue, making classification/grading unidentifiable; Lauren classification ‘Not applicable’ includes the aforementioned situations, as well as some non-Lauren type gastric cancers (e.g., neuroendocrine carcinoma); *Other rare tumors: 3 cases of EBV-associated gastric cancer, 2 cases of EBV-associated tubular adenocarcinoma (1 each in training and internal validation cohorts), 1 case of EBV-associated medullary carcinoma with lymphoid stroma (training cohort). “*PCC, poorly cohesive carcinoma; SRCC, signet ring cell carcinoma; pCR, pathological complete regression; MiNEN, mixed neuroendocrine non-neuroendocrine neoplasms; TRG, tumor regression grade.

### 1.4 Statistical methods

Data analysis was performed using GraphPad Prism 10 and R software (R version 4.5). Normally distributed continuous variables were expressed as x ± s, and t-tests were used for intergroup comparisons. Non-normally distributed continuous variables were expressed as median [M (range)]. Categorical variables were expressed as n (%), and intergroup comparisons used the χ² test or Fisher’s exact test. Linear regression analysis is used to examine the linear relationship between two continuous variables. Survival curves were plotted using the Kaplan-Meier (K-M) method, and the log-rank test was used for intergroup comparisons. COX regression analysis was used for OS and PFS prognostic risk factors. A total of 227 patients from Nanjing Drum Tower Hospital were randomly assigned in a 7:3 ratio to a training cohort and an internal validation cohort (The training cohort is used for model training), with details in **Table 1**. Variables with P<0.05 in univariate analysis were included in multivariate analysis. P<0.05 was considered statistically significant. Independent risk factors were introduced into R software to construct a clinical nomogram prediction model. Receiver operating characteristic (ROC) curves were plotted, and the area under the curve (AUC) was calculated. An AUC > 0.75 was considered to indicate good predictive ability of the model. Calibration curves of the nomogram model’s predicted probabilities versus actual complication rates were plotted to verify the consistency of the nomogram prediction model. Decision curve analysis (DCA) was performed to evaluate the clinical utility of the prediction model by quantifying the net benefit (NB) across a range of threshold probabilities. The number of dissected lymph nodes was incorporated into the aforementioned COX analysis using restricted cubic spline (RCS). An overall P-value and a nonlinear P-value both <0.05 indicated a significant nonlinear correlation.

## 2 Results

### 2.1 Baseline data

A total of 287 patients were enrolled, including 227 from Nanjing Drum Tower Hospital (August 2018 to December 2024) as primary center cohort and an external validation cohort of 60 patients (15 from Peking Union Medical College Hospital, 20 from Beijing Friendship Hospital affiliated with Capital Medical University, and 25 from the Seventh Medical Center of the Chinese PLA General Hospital). Among all 287 patients,47.74% had a favorable treatment response (TRG0 and TRG1), with 22.30% (64 cases) achieving pathological complete remission (pCR) of the primary tumor; however, 4 of these 64 patients still had lymph node metastasis (ypT0N+). There were no statistically significant differences in baseline data between the training cohort (n=158), internal validation cohort (n=69) and external validation cohort (n=60) (P>0.05) as shown in **Table 1**.

### 2.2 Risk factor analysis for OS and PFS after CICT

Univariate COX regression analysis showed that age (P = 0.013), hypertension (P<0.001), poorly cohesive carcinoma (PCC, including signet ring cell carcinoma [SRCC]) (P<0.001), mixed adenocarcinoma (P = 0.004), TRG3 (P = 0.011), AJCC Stage III (P = 0.005), tumor/tumor bed ≥5cm (P<0.001), ypT4 (P<0.001), lymph node metastasis (ypN+) (P<0.001), diffuse Lauren type (P = 0.001), mixed Lauren type (P = 0.007), poor differentiation (P = 0.003), vascular invasion (P<0.001), and neural invasion (P<0.001) were risk factors for OS (**Table 2**). Multivariate COX regression analysis identified age (P = 0.004), hypertension (P = 0.010), PCC (including SRCC) (P = 0.003), lymph node metastasis (P = 0.030), and neural invasion (P = 0.007) as independent risk factors for OS.

**Table 2:** Univariate and multivariate COX regression analysis of overall survival (OS).

Variable	Univariate					Multivariate				
	β	S.E	Z	*P*	HR (95% CI)	β	S.E	Z	*P*	HR (95% CI)
Gender (Female vs Male)	0.04	0.37	0.10	0.923	1.04 (0.51 ~ 2.12)					
Age/years	0.04	0.02	2.47	**0.013**	1.04 (1.01 ~ 1.07)	0.05	0.02	2.90	**0.004**	1.05 (1.02 ~ 1.09)
BMI/Kg·m-2	-0.07	0.05	-1.36	0.173	0.93 (0.84 ~ 1.03)					
Diabetes (Yes vs No)	0.02	0.35	0.06	0.950	1.02 (0.51 ~ 2.04)					
Hypertension (Yes vs No)	1.07	0.28	3.77	**<.001**	2.91 (1.67 ~ 5.08)	0.81	0.31	2.56	**0.010**	2.24 (1.21 ~ 4.16)
Surgical method (Lap vs Open)	-0.05	0.52	-0.09	0.927	0.95 (0.34 ~ 2.66)					
Intraoperative blood loss/ml	0.00	0.00	0.50	0.618	1.00 (1.00 ~ 1.00)					
Operation time/min	0.00	0.00	1.11	0.265	1.00 (1.00 ~ 1.01)					
Tumor/Tumor bed location										
Middle					1.00 (Ref)					
Upper	-0.28	0.33	-0.83	0.405	0.76 (0.40 ~ 1.45)					
Lower	-0.34	0.48	-0.69	0.487	0.71 (0.28 ~ 1.84)					
Upper-Middle	0.83	0.46	1.80	0.072	2.30 (0.93 ~ 5.69)					
Middle-Lower	-0.27	0.63	-0.42	0.674	0.77 (0.22 ~ 2.65)					
Total	-15.25	4129.62	-0.00	0.997	0.00 (0.00 ~ Inf)					
Histological type										
Not applicable*					1.00 (Ref)					1.00 (Ref)
Tubular adenocarcinoma	0.62	0.44	1.40	0.161	1.86 (0.78 ~ 4.42)	-0.29	0.49	-0.59	0.556	0.75 (0.28 ~ 1.97)
PCC (incl. SRCC) *	2.04	0.46	4.39	**<.001**	7.68 (3.09 ~ 19.10)	1.55	0.51	3.01	**0.003**	4.71 (1.72 ~ 12.88)
Mixed adenocarcinoma	1.48	0.52	2.85	**0.004**	4.41 (1.59 ~ 12.23)	0.06	0.61	0.11	0.915	1.07 (0.33 ~ 3.50)
Other rare tumors	0.68	0.59	1.16	0.245	1.98 (0.63 ~ 6.24)	-0.25	0.63	-0.40	0.686	0.78 (0.23 ~ 2.66)
TRG*										
0					1.00 (Ref)					
1	0.70	0.58	1.21	0.227	2.01 (0.65 ~ 6.25)					
2	1.06	0.55	1.91	0.056	2.89 (0.97 ~ 8.56)					
3	1.39	0.55	2.54	**0.011**	4.02 (1.37 ~ 11.80)					
AJCC Tumor Stage										
pCR					1.00 (Ref)					
I	-0.40	0.76	-0.53	0.599	0.67 (0.15 ~ 2.99)					
II	0.72	0.57	1.27	0.202	2.06 (0.68 ~ 6.30)					
III	1.49	0.53	2.81	**0.005**	4.43 (1.57 ~ 12.54)					
ypT0N+	-14.36	2806.00	-0.11	0.996	0.00 (0.00 ~ Inf)					
Tumor/tumor bed max. diameter (≥5cm vs <5cm)	1.00	0.27	3.64	**<.001**	2.72 (1.59 ~ 4.66)					
T stage										
ypT0					1.00 (Ref)					
ypT1	-0.03	0.76	-0.03	0.974	0.97 (0.22 ~ 4.36)					
ypT2	0.96	0.61	1.57	0.116	2.62 (0.79 ~ 8.74)					
ypT3	0.95	0.54	1.76	0.078	2.59 (0.90 ~ 7.46)					
ypT4	2.01	0.57	3.52	**<.001**	7.43 (2.43 ~ 22.71)					
Lymph node metastasis (Yes vs No)	1.32	0.31	4.25	**<.001**	3.75 (2.04 ~ 6.91)	0.78	0.36	2.17	**0.030**	2.19 (1.08 ~ 4.42)
Lauren classification										
Not applicable*					1.00 (Ref)					
Intestinal	0.42	0.37	1.16	0.247	1.53 (0.75 ~ 3.13)					
Diffuse	1.32	0.40	3.27	**0.001**	3.76 (1.70 ~ 8.30)					
Mixed	1.19	0.44	2.68	**0.007**	3.29 (1.38 ~ 7.87)					
Differentiation grade										
Not applicable					1.00 (Ref)					
G1	-0.04	0.82	-0.05	0.957	0.96 (0.19 ~ 4.79)					
G2	0.80	0.47	1.72	0.086	2.24 (0.89 ~ 5.60)					
G3	1.34	0.45	2.95	**0.003**	3.80 (1.57 ~ 9.22)					
Vascular invasion (Yes vs No)	1.20	0.27	4.39	**<.001**	3.32 (1.94 ~ 5.68)					
Neural invasion (Yes vs No)	1.34	0.29	4.62	**<.001**	3.81 (2.16 ~ 6.73)	0.92	0.34	2.69	**0.007**	2.50 (1.28 ~ 4.87)

HR, Hazard Ratio; CI, Confidence Interval; *Other abbreviations and notes same as **Table 1**. The values with P < 0.05 were highlighted in bold.

For PFS, univariate COX regression analysis showed that hypertension (P = 0.002), upper-middle stomach location (P = 0.019), PCC (including SRCC) (P<0.001), mixed adenocarcinoma (P = 0.003), TRG3 (P<0.001), AJCC Stage III (P<0.001), tumor/tumor bed ≥5cm (P = 0.006), ypT4 (P<0.001), lymph node metastasis (P<0.001), diffuse Lauren type (P = 0.046), mixed Lauren type (P = 0.002), poor differentiation (P = 0.030), vascular invasion (P<0.001), and neural invasion (P<0.001) were risk factors (**Table 3**). Multivariate analysis confirmed hypertension (P<0.001), PCC (including SRCC) (P = 0.008), lymph node metastasis (P = 0.002), and vascular invasion (P = 0.020) as independent risk factors for PFS.

**Table 3:** Univariate and multivariate COX regression analysis of progression-free survival (PFS).

Variable	Univariate					Multivariate				
	β	S.E	Z	*P*	HR (95%CI)	β	S.E	Z	*P*	HR (95%CI)
Gender (Female vs Male)	-0.18	0.31	-0.56	0.572	0.84 (0.46 ~ 1.54)					
Age/years	0.01	0.01	0.75	0.451	1.01 (0.98 ~ 1.04)					
BMI/Kg·m-2	-0.01	0.04	-0.24	0.806	0.99 (0.91 ~ 1.08)					
Diabetes (Yes vs No)	0.33	0.31	1.05	0.293	1.39 (0.75 ~ 2.55)					
Hypertension (Yes vs No)	0.82	0.26	3.09	**0.002**	2.26 (1.35 ~ 3.79)	1.00	0.29	3.41	**<.001**	2.73 (1.53 ~ 4.86)
Surgical method (Lap vs Open)	0.39	0.38	1.02	0.310	1.47 (0.70 ~ 3.10)					
Intraoperative blood loss/ml	0.00	0.00	1.60	0.110	1.00 (1.00 ~ 1.00)					
Operation time/min	0.00	0.00	1.94	0.053	1.00 (1.00 ~ 1.01)					
Tumor/Tumor bed location										
Middle					1.00 (Reference)					
Upper	0.03	0.41	0.08	0.935	1.03 (0.46 ~ 2.32)					
Lower	-0.15	0.31	-0.49	0.623	0.86 (0.47 ~ 1.57)					
Upper-Middle	1.05	0.45	2.34	**0.019**	2.87 (1.19 ~ 6.93)					
Middle-Lower	-15.22	2828.52	-0.01	0.996	0.00 (0.00 ~ Inf)					
Total	-0.48	0.63	-0.77	0.441	0.62 (0.18 ~ 2.11)					
Histological type										
Not applicable*					1.00 (Ref)					1.00 (Ref)
Tubular adenocarcinoma	0.48	0.36	1.32	0.188	1.62 (0.79 ~ 3.30)	-1.22	0.85	-1.42	0.154	0.30 (0.06 ~ 1.58)
PCC (incl. SRCC) *	1.48	0.40	3.69	**<.001**	4.38 (2.00 ~ 9.61)	1.49	0.56	2.67	**0.008**	4.42 (1.48 ~ 13.16)
Mixed adenocarcinoma	1.30	0.44	2.94	**0.003**	3.66 (1.54 ~ 8.70)	-1.19	0.97	-1.22	0.221	0.31 (0.05 ~ 2.04)
Other rare tumors	0.28	0.54	0.53	0.599	1.33 (0.46 ~ 3.82)	-1.00	0.82	-1.22	0.224	0.37 (0.07 ~ 1.84)
TRG*										
0					1.00 (Ref)					
1	0.37	0.46	0.81	0.420	1.45 (0.59 ~ 3.60)					
2	0.61	0.45	1.36	0.175	1.84 (0.76 ~ 4.44)					
3	1.43	0.43	3.35	**<.001**	4.19 (1.81 ~ 9.69)					
AJCC Tumor Stage										
pCR					1.00 (Ref)					
I	-0.93	0.69	-1.35	0.177	0.39 (0.10 ~ 1.52)					
II	0.26	0.46	0.56	0.576	1.30 (0.52 ~ 3.22)					
III	1.38	0.41	3.36	**<.001**	3.97 (1.77 ~ 8.88)					
ypT0N+	-15.77	3489.30	-0.00	0.996	0.00 (0.00 ~ Inf)					
Tumor/tumor bed max. diameter (≥5cm vs <5cm)	0.70	0.25	2.76	**0.006**	2.02 (1.23 ~ 3.33)					
T stage										
ypT0					1.00 (Ref)					
ypT1	-0.28	0.63	-0.45	0.652	0.75 (0.22 ~ 2.57)					
ypT2	0.27	0.56	0.49	0.624	1.31 (0.44 ~ 3.91)					
ypT3	0.78	0.42	1.85	0.064	2.18 (0.96 ~ 4.95)					
ypT4	1.98	0.46	4.36	**<.001**	7.28 (2.98 ~ 17.76)					
Lymph node metastasis (Yes vs No)	1.47	0.28	5.16	**<.001**	4.34 (2.48 ~ 7.58)	1.02	0.33	3.06	**0.002**	2.78 (1.44 ~ 5.35)
Lauren classification										
Not applicable*					1.00 (Ref)					1.00 (Ref)
Intestinal	0.41	0.32	1.29	0.198	1.51 (0.81 ~ 2.83)	1.15	0.81	1.43	0.154	3.15 (0.65 ~ 15.28)
Diffuse	0.76	0.38	1.99	**0.046**	2.15 (1.01 ~ 4.56)	-0.78	0.53	-1.47	0.142	0.46 (0.16 ~ 1.30)
Mixed	1.19	0.38	3.09	**0.002**	3.28 (1.54 ~ 6.98)	1.37	0.91	1.50	0.133	3.94 (0.66 ~ 23.49)
Differentiation grade										
Not applicable					1.00 (Ref)					
G1	0.16	0.65	0.24	0.807	1.17 (0.33 ~ 4.20)					
G2	0.51	0.37	1.40	0.163	1.67 (0.81 ~ 3.43)					
G3	0.77	0.36	2.17	**0.030**	2.17 (1.08 ~ 4.36)					
Vascular invasion (Yes vs No)	1.14	0.25	4.57	**<.001**	3.13 (1.92 ~ 5.10)	0.69	0.30	2.32	**0.020**	2.00 (1.11 ~ 3.58)
Neural invasion (Yes vs No)	1.21	0.26	4.70	**<.001**	3.35 (2.02 ~ 5.56)					

HR, Hazard Ratio, CI, Confidence Interval; *Other abbreviations and notes same as **Table 1**. The values with P < 0.05 were highlighted in bold.

### 2.3 Establishment and evaluation of OS and PFS survival prediction models after conversion immunochemotherapy

**Figure 1:**
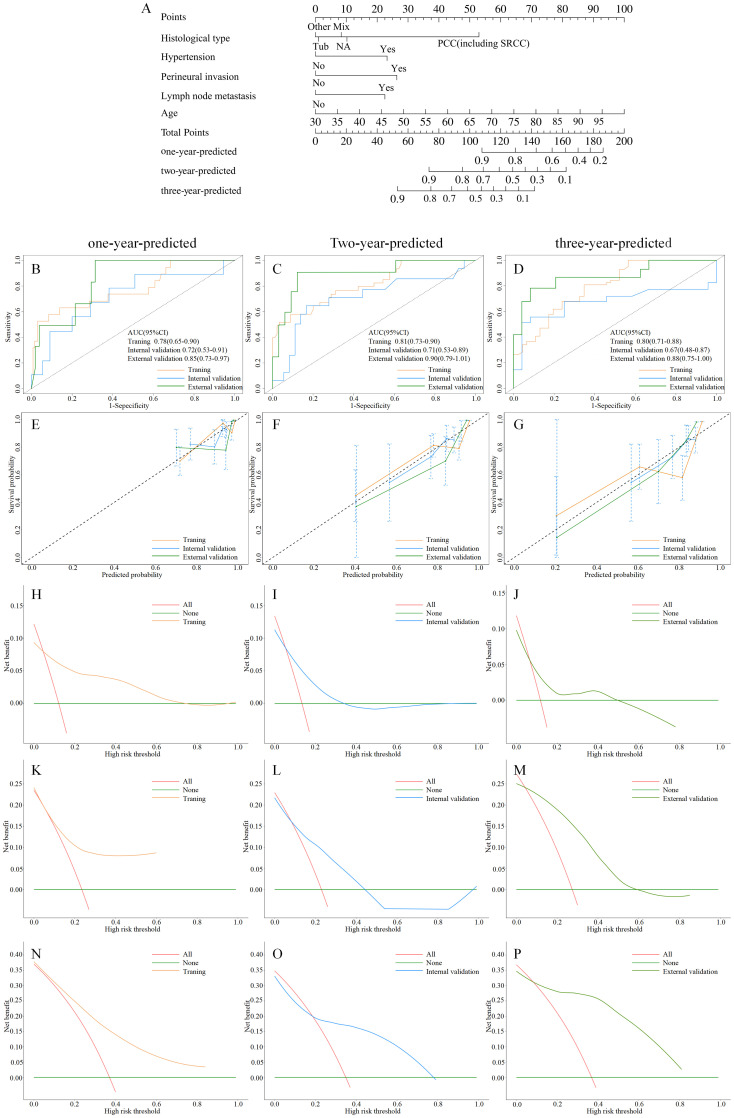
Nomogram **(A)** with points for prognostic variables including histological type, hypertension, perineural invasion, lymph node metastasis, and age predicts one-, two-, and three-year survival rates for overall survival (OS). Receiver operating characteristic (ROC) curves **(B–D)** compare training, internal validation, and external validation groups for predicted survival at one, two, and three years, reporting area under the curve (AUC) values. Calibration plots **(E–G)** assess agreement between predicted and actual survival probabilities. Decision curve analyses **(H–P)** display net benefit across high-risk thresholds for training, internal, and external validation datasets at each time point.

**Figure 2:**
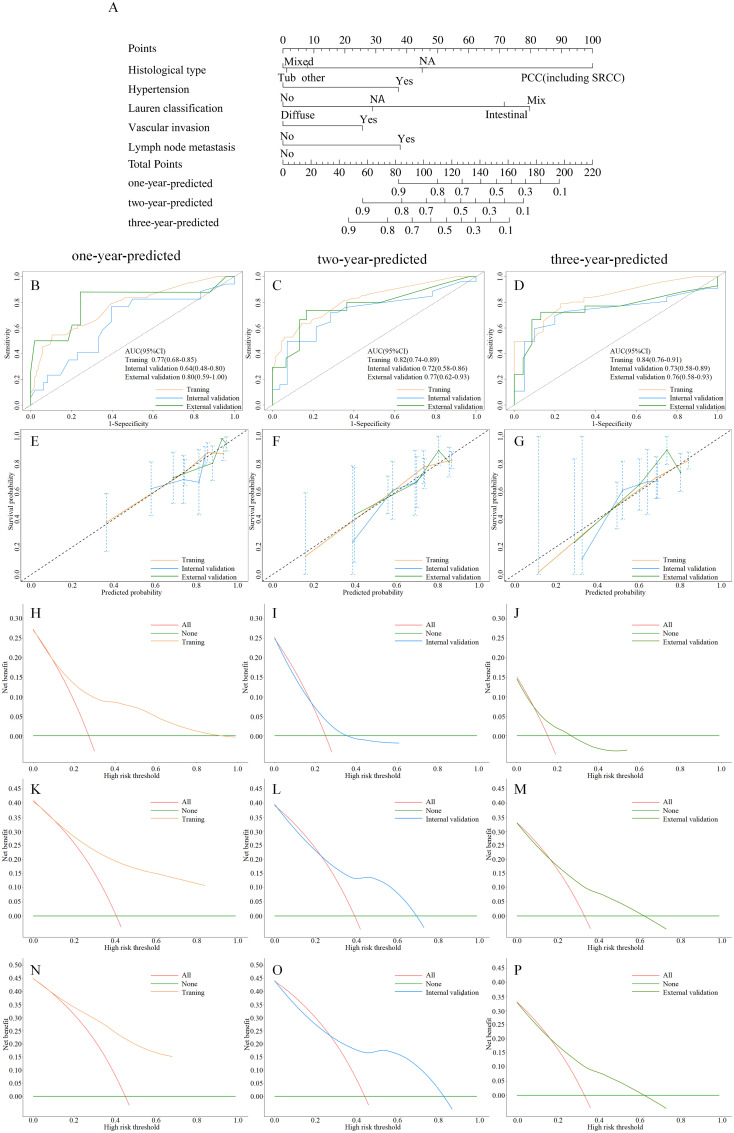
Nomogram **(A)** with points for prognostic variables including histological type, hypertension, Lauren classification, vascular invasion, lymph node metastasis predicts one-, two-, and three-year survival rates for progression-free survival (PFS). Receiver operating characteristic (ROC) curves **(B–D)** compare training, internal validation, and external validation groups for predicted survival at one, two, and three years, reporting area under the curve (AUC) values. Calibration plots **(E–G)** assess agreement between predicted and actual survival probabilities. Decision curve analyses **(H–P)** display net benefit across high-risk thresholds for training, internal, and external validation datasets at each time point.

OS and PFS prediction models were constructed based on independent risk factors (**Figures 1**, **2A**). For the OS nomogram, the 1-year, 2-year, and 3-year AUC values were 0.78 (95% CI: 0.65~0.90), 0.81 (95% CI: 0.73~0.90), and 0.80 (95% CI: 0.71~0.88) in the training validation; 0.72 (95% CI: 0.53~0.91), 0.71 (95% CI: 0.53~0.89), and 0.67 (95% CI: 0.48~0.87) in the internal validation cohort; and 0.85 (95% CI: 0.73~0.97), 0.90 (95% CI: 0.79~1.01), and 0.88 (95% CI: 0.75~1.00) in the external validation cohort (**Figure 1B**).

For the PFS nomogram, the 1-year, 2-year, and 3-year AUC values were 0.77 (95% CI: 0.68~0.85), 0.82 (95% CI: 0.74~0.89), and 0.84 (95% CI: 0.76~0.91) in the training cohort; 0.64 (95% CI: 0.48~0.80), 0.72 (95% CI: 0.58~0.86) and 0.73 (95% CI: 0.58~0.89), in the internal validation cohort; and 0.76 (95% CI: 0.58~0.93), 0.80 (95% CI: 0.59~1.00), and 0.77 (95% CI: 0.62~0.93) in the external validation cohort (**Figure 2B**). Calibration curves showed good overlap with standard curves, indicating high consistency between predicted and observed OS/PFS (**Figures 1E**, **2E–G**). Decision curve analysis (DCA) was conducted to evaluate the clinical utility of the prediction model of OS/PFS. The x-axis represents the threshold probability for intervention, and the y-axis represents the net benefit. (**Figures 1I**, **Figure 2I**).

### 2.4 Number of dissected lymph nodes and prognosis

**Figure 3:**
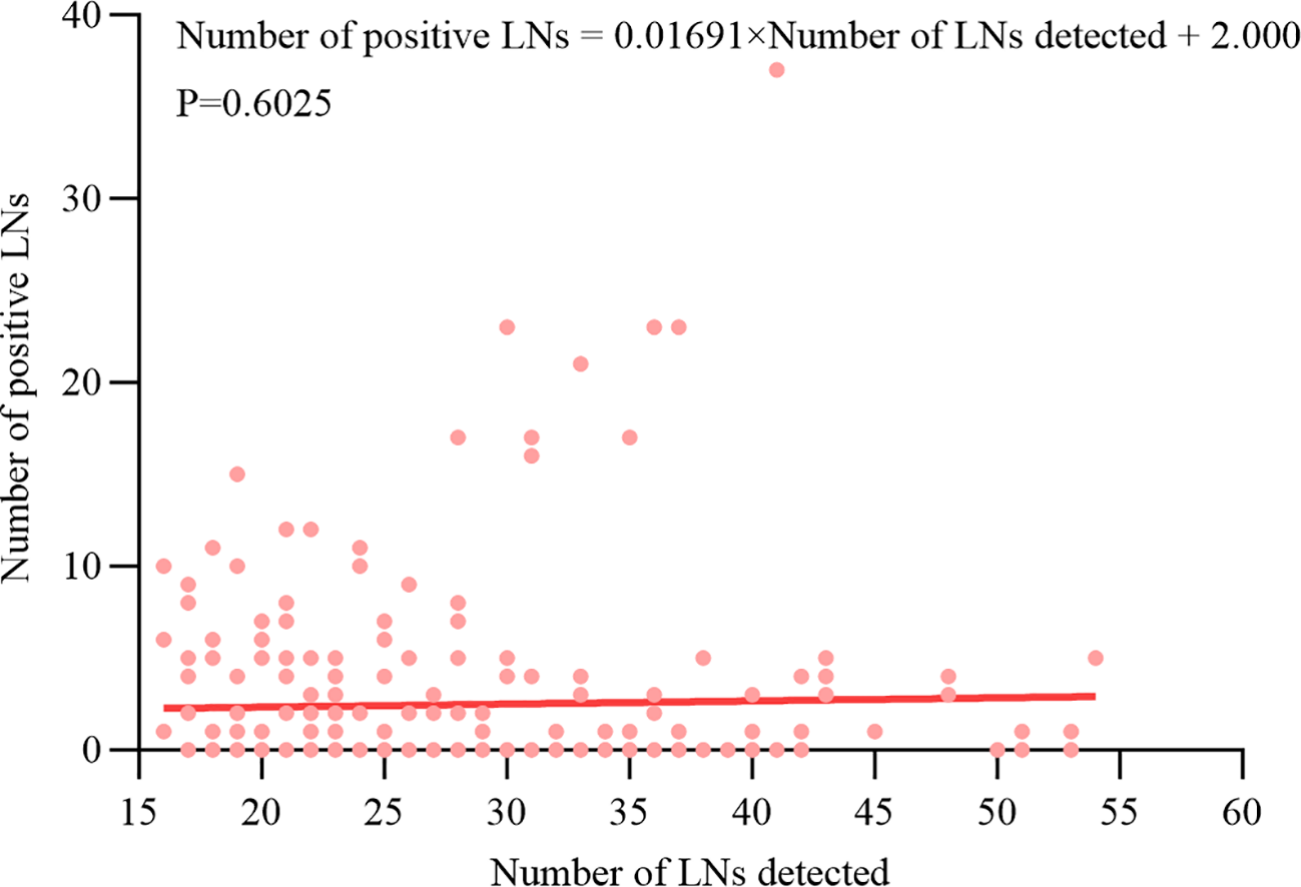
Scatter plot showing the relationship between number of lymph nodes detected and number of positive lymph nodes, with a linear regression line, regression equation, and P-value equal to 0.6025.

**Figure 4:**
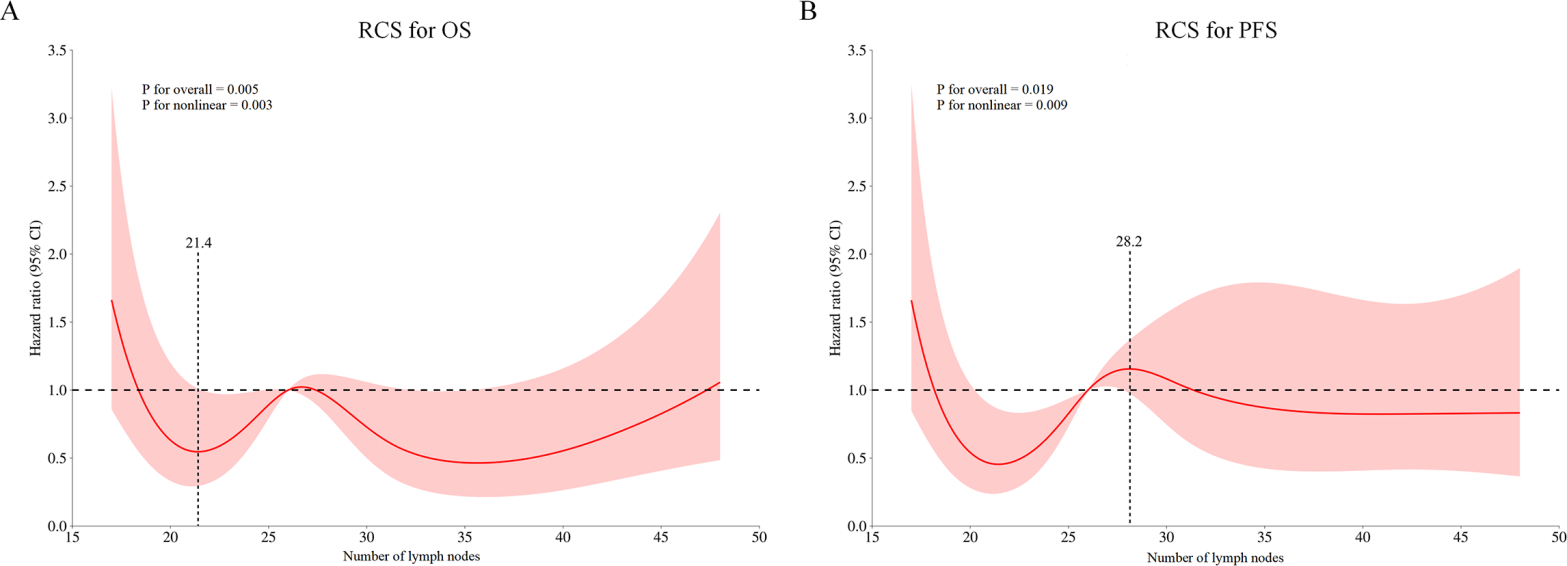
Plot RCS curves based on the number of lymph node dissections. **(A)** RCS curve of OS based on the number of lymph nodes dissected; **(B)** RCS curve of PFS based on the number of lymph nodes dissections. (RCS, restricted cubic spline; OS, overall survival; PFS, progression-free survival; CI, Confidence interval).

**Figure 5:**
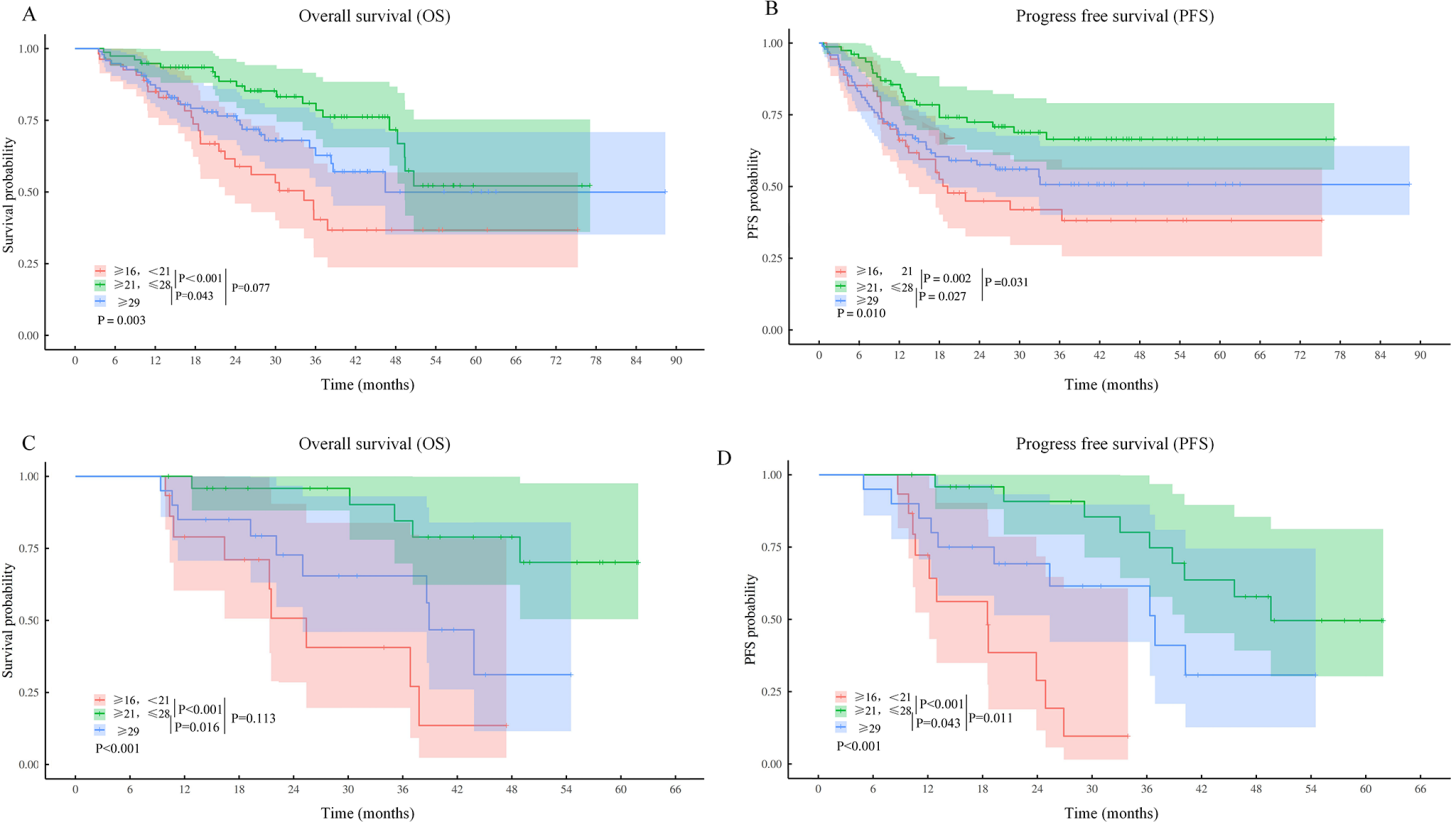
K-M curves of OS and PFS for patients with different numbers of lymph node dissections: **(A, B)** OS and PFS according to the number of dissected of 227 patients of primary center cohort; **(C, D)** OS and PFS according to the number of dissected of 60 patients of external validation cohort. (K-M curves, Kaplan-Meier curves; OS, overall survival, PFS, progression-free survival).

Correlation analysis showed no significant association between the number of positive lymph nodes and total dissected lymph nodes (P = 0.6025) (**Figure 3**). RCS analysis of the number of dissected lymph nodes (from 227 patients at Nanjing Drum Tower Hospital) revealed inflection points in HR values for OS and PFS at 21 and 28 lymph nodes (**Figure 4**). Patients were thus divided into three groups: <21, 21-28, and >28 lymph nodes dissected. In the group fewer than 21 lymph nodes dissected, the median overall survival (OS) was 34.27 months (95% CI: 22.43–NA); in the group with more than 28 lymph nodes dissected, the median OS was 46.43 months (95% CI: 49.33–NA); in the group with 21–28 lymph nodes removed, the median survival time had not yet been reached by the end of follow-up (NA),as less than 50% of patients had died. For PFS, patients with fewer than 21 resected lesions had a median PFS of 19.23 months (95% CI: 13.40–NA), whereas patients with more than 28 resected lesions or between 21 and 28 resected lesions had not reached median PFS at the time of follow-up. K-M survival analysis showed that patients with 21–28 lymph nodes dissected had significantly better OS and PFS than those with <21 (P<0.001 and P = 0.002) and >28 (P = 0.043 and P = 0.027) lymph nodes dissected (**Figure 5A**). Additionally, patients with >28 lymph nodes dissected had better PFS than those with <21 (P = 0.031), and a trend toward better OS (P = 0.077). Consistent trends were observed in the external validation cohort (**Figure 5C**).

## Discussion

Traditional perspectives emphasize dissecting ≥16 lymph nodes (preferably >30) to improve staging accuracy and eliminate micro-metastases, thereby enhancing patients’ survival (4). However, with evolving treatment strategies and deeper mechanistic understanding, traditional lymph node dissection has faced new challenges (6). As a key site for immune responses, lymph node dissection strategies significantly impact gastric cancer treatment efficacy. Therefore, this study, while retaining the traditional N staging requirement (number of dissected lymph nodes ≥16), conducted a retrospective analysis of LAGC patients at our hospital who received CICT followed by total gastrectomy and D2 lymph node dissection. Through univariate and multivariate COX regression analysis, we found that lymph node metastasis (ypN+) after conversion immunochemotherapy was an independent risk factor for OS and PFS ((P<0.001, P = 0.002). At the same time, our results also pointed out that poorly cohesive carcinoma (including signet ring cell carcinoma), hypertension, vascular invasion, and neural invasion are also independent risk factors for OS and/or PFS. Based on these, survival-related prediction models were established, and their 1-year, 2-year, and 3-year ROC curve areas were all greater than 0.75, showing a good model performance. Secondly, this study determined the threshold values for lymph node dissection as 21 and 28 through RCS analysis of the COX regression, and stratified analysis was performed based on these results. The OS and PFS-related KM curves both showed that patients with 21–28 dissected lymph nodes had the best OS and PFS. This result suggests that an insufficient or excessive number of dissected nodes may affect the efficacy of surgery after conversion immunochemotherapy, but the survival of those with excessive dissection is still better than those with insufficient dissection. This study is the first to investigate the effect of the number of dissected lymph nodes on the prognosis of LAGC patients received CICT, providing real-world evidence for the formulation of related guidelines in the future.

The metastatic status of lymph nodes is crucial for patients’ prognosis (14), lymph node colonization by tumors is often a sign of distant metastasis (15), hence patients with lymph node metastasis tend to have a poorer prognosis. Among resectable lung cancer patients receiving neoadjuvant immunotherapy, ypN+ is also a risk factor for postoperative mediastinal lymph node recurrence and metastasis, and this progresses with advancing N stage (16), which is consistent with the results of this study. Patients with ypN+ after CICT combined with surgery had worse OS and PFS compared to ypN0 patients. Therefore, sufficient lymph node dissection is necessary for a comprehensive assessment of lymph node metastasis and to minimize micro-metastasis. However, in the context of immunotherapy, excessive lymph node dissection may, on the contrary, reduce the efficacy of immunotherapy (8, 10). As an important site for initiating immune responses, the integrity of lymph node function is very important for patients’ prognosis and treatment benefits. Antigen encounters in the tumor drive local immune cell differentiation and residency, but long-term, repeated antigen stimulation and the tumor inflammatory environment eventually lead to the exhaustion of anti-tumor lymphocytes. Multiple studies have found that tumor-infiltrating lymphocytes share the same clonal atlas as T cells in tumor-draining lymph nodes, indicating that LNs play a continuous role in exporting anti-tumor-active immune cells (5, 17–19). The composition of immune cell populations within tumor lymph nodes, or the proportion of different immune cell subtypes, may also affect the benefits of immunotherapy. Existing research shows that a higher number of tissue-resident memory T cells in lymph nodes is associated with a better prognosis (3). Moreover, after anti-PD-L1 immunotherapy in non-metastatic lymph nodes, CD8+Tpex transforms into an activated state of intermediate-exhausted CD8 T cells (CD8+Tex-int). This lymph node response exhibits simultaneity and consistency with the circulating pool (5), indicating coordination between local immunity in the lymph nodes and systemic immunity. Furthermore, targeting ICIs to draining lymph nodes may yield better benefits than traditional systemic administration (20–22). Studies have also shown that combining T-cell immunoglobulin and mucin-domain containing molecule 3 (TIM-3) ligand galectin-9 inhibition during epidermal growth factor receptor (EGFR) targeted (tyrosine kinase inhibitor [TKI]) therapies in lung cancer patients can promote dendritic cell accumulation within tumor-draining lymph nodes (TDLNs) and enhance CD8^+^ T cell responses in the tumor microenvironment (23);CD86 blockade can also suppress effector regulatory T cell (Treg) responses, thereby enriching CD80+PD-L1+dendritic cells (DCs) and promoting cytotoxic T cell responses (24);Furthermore, the maintenance of conventional type 1 DCs (cDC1) is conducive to the maintenance of T-cell factor-1 (TCF-1) +CD8+T cells within draining lymph nodes; reduction of cDC1 increases the risk of antitumor immunotherapy failure (25), recruitment of TCF-1^+^CD8^+^ T cells—cells with active immune responsiveness—into the tumor microenvironment (TME) enhances the efficacy of ICIs (26), whereas impaired stimulation or failure of these cells to traffic to the TME diminishes the benefit of immunotherapy (27).In addition, under immune pressure, head and neck squamous cell carcinoma (HNSCC) cells promote the formation of immunosuppressive TDLNs via neuro-immune pathways, finally by reducing CCL5 secretion, they promote M2-like polarization of tumor-associated macrophages, thereby facilitating tumor growth and reducing the efficacy of ICIs (28). Moreover, efficient induction of CD169+ macrophages within draining lymph nodes can activate CD8^+^ T cells to exert antitumor immune activity and enhance the therapeutic effects of ICIs (29). Our study shows that for LAGC patients received CICT, sufficient lymph node dissection is essential. Currently, lymph node tracing techniques such as nanocarbon or indocyanine green have greatly improved the precision and quantity of lymph node dissection in gastric cancer, against this backdrop, controlling the extent of lymph node dissection has become a new challenge. For patients with LAGC patients received CICT, lymph node dissection requires maintaining an appropriate range; for those undergoing CICT, alterations in some lymph nodes—such as fusion, fibrosis or calcification—can increase the difficulty of detecting lymph nodes (30, 31); Furthermore, might alterations in tumors—such as treatment-related alterations or residual vascular invasion—further affect the visualisation of the lymph node tracer? Does the use of different administration methods in these patients influence the assessment of lymph node metastasis status? Similarly, after receiving CICT, could the immune characteristics within negative lymph nodes or persistently positive lymph nodes—whether immunosuppression-related markers (such as Treg, PD-L1, etc.) or markers associated with immune responsiveness (such as TCF-1^+^CD8^+^ T cells and CD8^+^ Tpex cells as mentioned above)—serve as factors of CICT response or resistance in gastric cancer patients, or as predictors of prognosis for this patient population? These remain issues that require further exploration and refinement at this stage.

For LAGC patients received CICT, adequate lymph node dissection is essential. Advanced lymph node tracing techniques (e.g., nanocarbon, indocyanine green) have significantly improved the precision and quantity of lymph node dissection in gastric cancer. However, controlling dissection extent has emerged as a new challenge. Reactive changes in lymph nodes and tumor beds after CICT, as well as residual vascular invasion, may affect tracer imaging. Additionally, different drug delivery methods may influence the assessment of lymph node metastatic status — issues requiring further exploration and optimization.

This study has several limitations. First, as a retrospective study, it is subject to selection bias. Second, while the prognostic cohort model underwent internal validation and external validation, the total sample size still needs to be enlarged, the long-term survival outcome is lacking. On the same basis as the present study, future efforts should further investigate and clarify how the immune microenvironment within lymph nodes of patients receiving CICT influences ICIs response and clinical benefit, and to explore the mechanisms underlying lymph node responses to ICIs in gastric cancer patients.

In summary, lymph node metastasis is an independent survival risk factor for LAGC patients received CICT. Our finding that 22.47% of patients had <50% tumor regression suggests that lymph node-targeted sensitized immunotherapy may improve survival benefits for this subgroup. Excessive lymph node dissection reduces CICT efficacy, and the optimal dissection range is recommended as 21-28. Due to inherent limitations, potential confounding factors could not be fully adjusted or balanced. Larger sample sizes and longer follow-up are needed in future studies.

## Data Availability

The original contributions presented in the study are included in the article. Further inquiries can be directed to the corresponding authors.
